# The effect of working memory training on situation awareness in a flight simulator

**DOI:** 10.1007/s10111-022-00707-1

**Published:** 2022-07-04

**Authors:** Yue Zhou, Di Wu, Chaoxian Wang, Kewei Sun, Pengbo Xu, Ziwei Wang, Wei Xiao

**Affiliations:** School of Military Medical Psychology, Air Force Medical University, Xi’an, 710032 China

**Keywords:** Situation awareness, 3D-SART, SAGAT, Working memory training, Dual N-back task

## Abstract

The close relationship between working memory and situation awareness (SA) has been confirmed and further empirical investigations are lacking. The main aim of this study was to demonstrate the feasibility of working memory training for improving SA. Thirty-eight participants completed a challenging flight scenario in a high-fidelity flight simulator and were randomized into a training group (*n* = 20) or a control group (*n* = 18). The training group engaged in an adaptive dual N-back task for 2 weeks, while the control group was given a negative control task. Three-dimensional situation awareness rating technique (3D-SART) scores and situation awareness global assessment technique (SAGAT) scores were recorded to evaluate pretest and posttest SA. The results showed that both situational understanding dimension scores in the 3D-SART and SAGAT scores were significantly increased from the pretest to the posttest in the training group, while the control group showed no significant differences. It was concluded that working memory training can effectively improve individuals’ SA, which has important implication for future research.

## Introduction

Situation awareness (SA) is one of the most researched topics in aviation safety, especially in human error research. Loss of SA has been identified as a major cause of aviation accidents (Kharoufah et al. 2018); notably, 88% of major air carrier incidents are at least partially attributable to problems with SA(Endsley, 1999). Because of the importance of SA for safety operations (Pritchett 2015), it has been widely used in transportation (Salmon et al. 2014), nuclear power plant monitoring (Carvalho et al. 2012), medical treatment (Tower et al. 2019), and other safety–critical domains. Endsley’s (1995a) definition, “the perception of elements in the environment within a volume of time and space; the comprehension of their meaning; and the projection of their status in the near future”, is widely accepted. Many studies have shown that people’s SA is influenced by mental workload, emotional states, cognitive capacities, level of expertise, and other factors (Cak et al. 2020; Jeon et al. 2014; Wanyan et al. 2018). However, little is known about whether a simple, practical training method can effectively improve SA.

Working memory, as a core cognitive ability, is closely related to SA. The information processing theoretical model proposed by Endsley (1995a) emphasizes that SA is closely related to some cognitive abilities such as attention, working memory, and long-term memory. Previous studies have also confirmed that working memory plays an important role in SA. Cak et al. (2020) reported a positive correlation and a predictive relationship between working memory capacity and SA. Sulistyawati et al. (2011) found that the information perception of pilots for Level 1 SA was closely related to spatial working memory and spatial reasoning ability. Johannsdottir and Herdman (2010) found that adding visuospatial and phonological working memory tasks to a driving simulation negatively affected the Level 2 (comprehension) SA of drivers with at least two years of driving experience. Gutzwiller and Clegg (2013) showed that working memory predicted the performance of novices in a firefighting task relying on Level 3 SA, which involves the projection of the near future. In complex operating environments, such as the cockpit of an aircraft, limited working memory capacity can make it difficult for pilots to acquire and integrate information from different sources in a timely manner, reducing SA (Ericsson and Delaney 1999). In other words, working memory is an important factor for individuals to acquire and maintain SA.

Fortunately, working memory is trainable, and working memory training can improve performance on related tasks (Klingberg et al. 2005) and can affect other cognitive abilities associated with working memory, such as attention control, reasoning, and fluid intelligence (Clark et al. 2017; Jaeggi et al. 2008). The dual N-back task is an advanced variant of the N-back task and is based on the basic principle of the dual task paradigm, which requires the simultaneous presentation of a standard auditory N-back task and a standard visual N-back task (Schmiedek et al. 2014). Since the dual N-back task includes simultaneous audio-visual dual channels, and two tasks that are relatively independent, it requires dual executive control, which is not conducive to the development of specialized strategies and the involvement of automated processing. Therefore, it is considered to be more effective in enhancing individuals' executive function abilities, binding processing and attention control than a single task(Li et al. 2021; Ørskov et al. 2021), and it is also more in line with the multitask operation requirements during an actual flight.

In light of the previous literature, a flight simulator was used as an experimental platform to investigate the effect of working memory training on SA. Two common SA measures, the 3D-SART (Selcon et al. 2001) and SAGAT (Endsley 2000), were used to assess subjective and objective SA, respectively. Observing the beneficial effect of working memory training on SA was the main critical aim of this research. If the method is verified and widely accepted, it will greatly advance research in the field of SA and will certainly provide a direction for future development.

## Methods

### Participants

A total of 38 participants from the Air Force were randomly divided into a training group (16 males and 4 females; mean age 19.65 ± 0.875 years) or a control group (14 males and 4 females; mean age 20.17 ± 0.857 years). There was no significant difference in sex or age between the two groups (*P* > 0.05). All subjects were 18 ~ 22 years old, physically and mentally healthy, were right-handed, had normal or corrected-normal vision, did not experience motion sickness or motion sickness symptoms and had not participated in other relevant psychological experiments. All subjects signed informed consent forms in accordance with the Declaration of Helsinki. The study was approved by the local ethics committee and strictly followed its recommendations.

### Apparatus

**Fig. 1:**
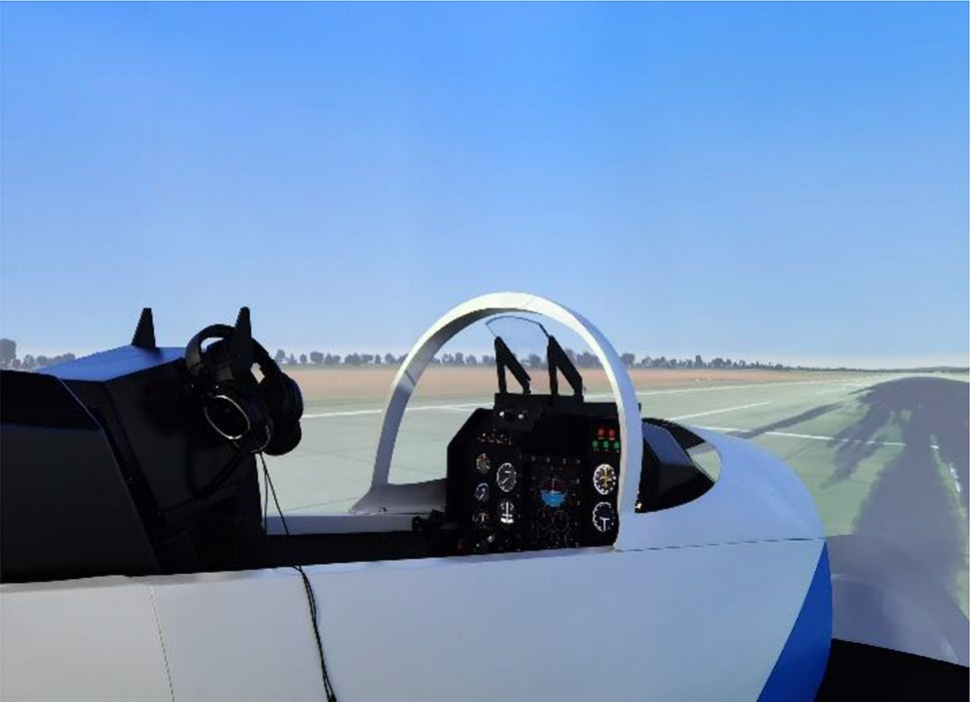
External view of the JL-8 high-fidelity flight simulator

The simulated flight was conducted using a JL-8 high-fidelity flight simulator (see Fig. 1). The simulator has a 220° spherical field, with a 200° horizontal view and 55° vertical view. The instructor panel allowed an external controller to adjust various functions, such as freezing the simulator, introducing malfunction scenarios, and changing the weather conditions. The center of the flight panel had a multifunction display (MFD), including a horizon indicator, an airspeed indicator, altimeter, and a vertical speed indicator; the left side of the flight panel had a turning sideslip indicator, an intake pressure indicator, a tachometer, an intake temperature indicator, etc.; and the right side of the flight panel had a radio compass, a gyrostabilized magnetic compass, a three-purpose indicator, etc.

## Materials

### Flight scenario

For SA measurements, a cognitively demanding 30- to 45-min flight scenario with 6 examination subjects was developed and verified in consultation with subject matter experts (SMEs), who each had an average of 3000 total flight hours. The flight instructor, also assigned as the air traffic controller (ATC), generated particular events for all participants at approximately the same time and location in the flight.

Before the experiment, flight experts conducted standardized flight training for all participants, including theory knowledge, system operation and fault handling, which was divided into theoretical lectures, cockpit internships and flight evaluations in three stages. At the beginning of the training, all participants were given enough time for an uneventful flight warm-up to ensure that they were proficient enough to begin the experiment.

During the flight, all participants were not only required to complete the examination according to the system instructions while flying the planned route, but also needed to address sudden malfunctions quickly, such as brake fault problems and bad weather; the participants also needed to answer the instructor’s questions immediately after the examination of each subject, including the extravehicular environment, the instruments in the cabin and identification and treatment of sudden malfunctions. As a result, each subject required a considerable amount of mental work to complete the examination, while also forcing participants to pay attention to environmental factors and sudden malfunctions. The research questions for the measurement of SA were extracted from the SA requirements during the flight scenario in consultation with the SMEs.

### Training task

For the training task, a task similar to that described by Jaeggi et al. (2007) was used, which was an adaptive dual N-back task where squares were presented randomly on a computer screen (appearing in all but the middle square of a 3 × 3 grid) at a rate of one square every 3 s (stimulus presentation time, 500 ms; interstimulus interval, 2500 ms). As each square was presented, one of eight numbers (integers from 1 to 9, excluding 5) was selected randomly and presented through earphones. A quick response was required whenever one of the presented stimuli matched a stimulus presented N positions back in the sequence. The value of N was the same for both streams of stimuli. For both squares and numbers, the ratio of consistent to inconsistent trials was 3:7, and all trials were presented randomly. Participants entered their responses manually by pressing the letter ‘‘A’’ on a standard keyboard with their left index finger for visual targets and pressing the letter ‘‘L’’ with their right index finger for auditory targets. No responses were required for nontargets.

To familiarize the participants with the rules and maintain their motivation during training, real-time feedback was provided each time they pressed a key to enter a response. After each block, the participants’ individual performances were analyzed, and in the subsequent block, the value of N was adapted accordingly: if the accuracy rate for a given modality was more than 0.85, the value of N increased by 1, whereas if the accuracy was less than 0.75, the value of N decreased by 1; in all other cases, N remained unchanged. Each training session included 20 blocks, with 20 + N trials per block, resulting in a daily training duration of 40 min. Each training session started at level 1, with a 60 s break between blocks.

### Raven’s advanced progressive matrices

Raven’s Advanced Progressive Matrices are often used to assess an individual’s fluid intelligence. Jaeggi et al. (2014) argued that, when the odd-numbered and even-numbered items are separated to form different versions of the test, the even-numbered version is more difficult than the odd-numbered version. Therefore, they divided the tests into more balanced categories: version A contained Items 3, 4, 5, 8, 9, 10, 15, 17, 18, 19, 20, 25, 27, 29, 31, 32, 33 and 34, and version B contained Items 1, 2, 6, 7, 11, 12, 13, 14, 16, 21, 22, 23, 24, 26, 28, 30, 35, 36 and 37. In addition, Jaeggi et al. found that without a time limit, most participants’ scores easily displayed a ceiling effect, thus masking the transfer effect. In this study, version A was used for the pretest, and version B was used for the posttest. The time allotted for each test was 8 min, and the number of correct answers was used as a measure of intelligence.

### 3D-SART

The 3D-SART is the most widely used subjective situational awareness assessment method. Vidulich (2000) performed a meta-analysis that verified its sensitivity, and Selcon et al. (2001) used computer simulation missions to verify its accuracy. After the participants completed the experiment, they subjectively evaluated the experimental situation. There were 10 items, representing the following three dimensions: attention demand (D), attention supply (S) and situational understanding (U). A participant’ score on this scale is calculated as SA = U − D + S; the higher the score is, the better the SA.

### SAGAT

SAGAT, first proposed by Endsley (2000), is one of the most effective and objective methods to quantify SA, measuring it directly during the experimental process. During the flight simulation experiment, the participants were asked questions related to the experimental situation; the results were recorded, and the accuracy of the responses was used to evaluate the participants’ SA. The test consisted of 38 paper-and-pencil queries administered in 6 batches of 5, 4, 8, 6, 8, and 7 questions, reflecting SA on the perception, comprehension, and projection levels. The simulation was frozen for no more than 5 min at 6 predefined freeze points after the end of each examination topic during the scenario. At the freeze points, the participants were required to turn to the right to mark their answers; this prevented them from seeing the flight displays while giving their answers. Only response accuracy values (not response latency value) were recorded; the accuracy values were combined into a single score by summing the total number of correct responses.

## Design and procedure

The training group performed the dual N-back task as their training task, which was presented using MATLAB 2018. All experiments started with a short practice phase to ensure that the participants fully understood the rules of the task. All training tasks were completed in the laboratory over a total of 4 weeks. The two groups were assessed with Raven’s Advanced Progressive Matrices to evaluate their pretest and posttest performance in fluid intelligence; the 3D-SART and SAGAT were used to evaluate SA performance before and after training in simulated flights at weeks 1 and 4. In weeks 2 and 3, the training group completed the training task for 40-min sessions, 6 times per week, while the control group was given a negative control task. Before the flight simulation, the participants completed Raven’s Advanced Progressive Matrices. During the flight simulation, questions for the SAGAT were asked at 6 preset freeze points, and the 3D-SART scale was completed immediately after the mission.

## Results

### Training results

**Fig. 2:**
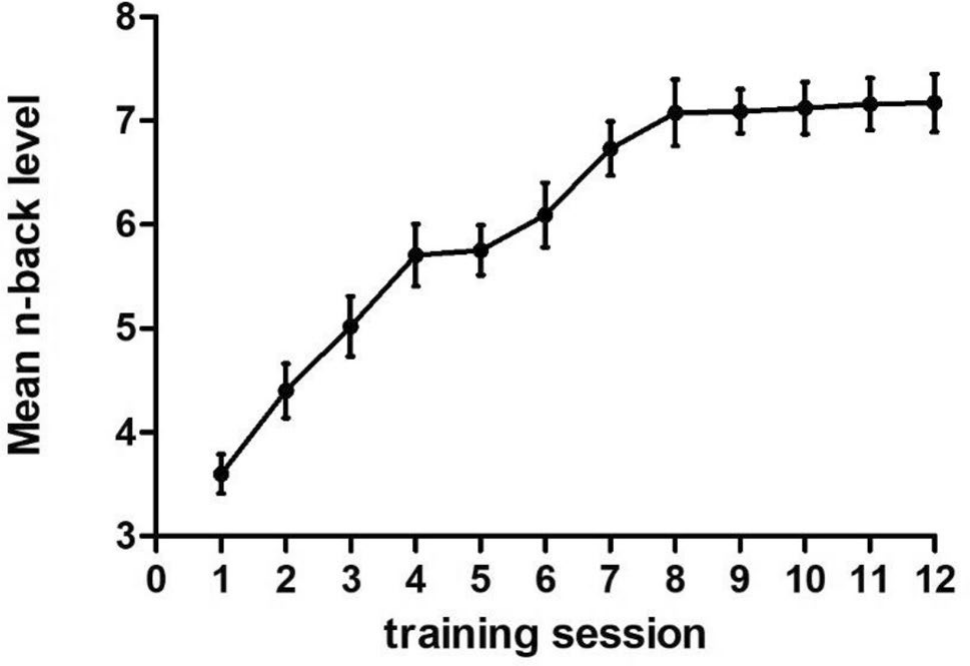
Simple line plot of the mean *N* value in the training group for the *N*-back task throughout the training sessions. Error bars indicate the SE

The mean *N* value of each training session was chosen as the performance for the training session. Repeated-measures ANOVAs were conducted to compare performance in the first session with performance in the twelfth session. As shown in Fig. 2, the mean *N* value for the task gradually increased from the first to the last training session, and this improvement was statistically significant [*F* (1, 19) = 260.71, *P* < 0.001, *η*^2^ = 0.93]. In the training group, the mean N level after the dual N-back task increased gradually with the number of training sessions and then became stable after the ninth training session.

### Raven’s advanced progressive matrices results

**Fig. 3:**
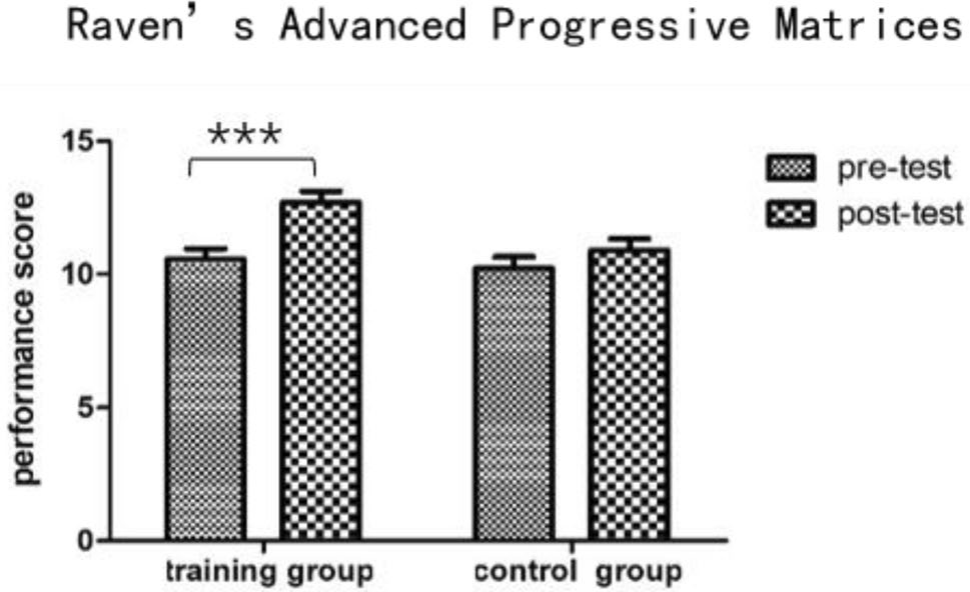
Performance on Raven’s advanced progressive matrices for each group on the pretest and posttest. Error bars indicate the SE

There was no significant difference between the two groups in terms of pretest scores on Raven’s Advanced Progressive Matrices [*t* (36) = 0.56, *P* = 0.581]. A 2 (control group vs. training group) × 2 (pretest vs. posttest) mixed-model ANOVA was performed for the scores of the two groups; the ANOVA identified significant main effects of group [*F* (1, 36) = 4.54, *P* = 0.040, *η*^2^ = 0.11] and time [*F* (1, 36) = 21.05, *P* < 0.001, *η*^2^ = 0.37] as well as an interaction effect of group and time [*F* (1, 36) = 5.84, *P* = 0.021, *η*^2^ = 0.14]. Then, the effects of the two factors were assessed through simple effect analysis. As shown in Fig. 3, the scores of the training group were significantly increased in the posttest compared with the pretest [*F* (1, 36) = 25.89, *P* < 0.001, *η*^2^ = 0.42], while there was no significant difference for the control group (*p* = 0.143). The results indicated that the fluid intelligence of the training group was significantly increased after training compared with before training.

### 3D-SART results

**Fig. 4:**
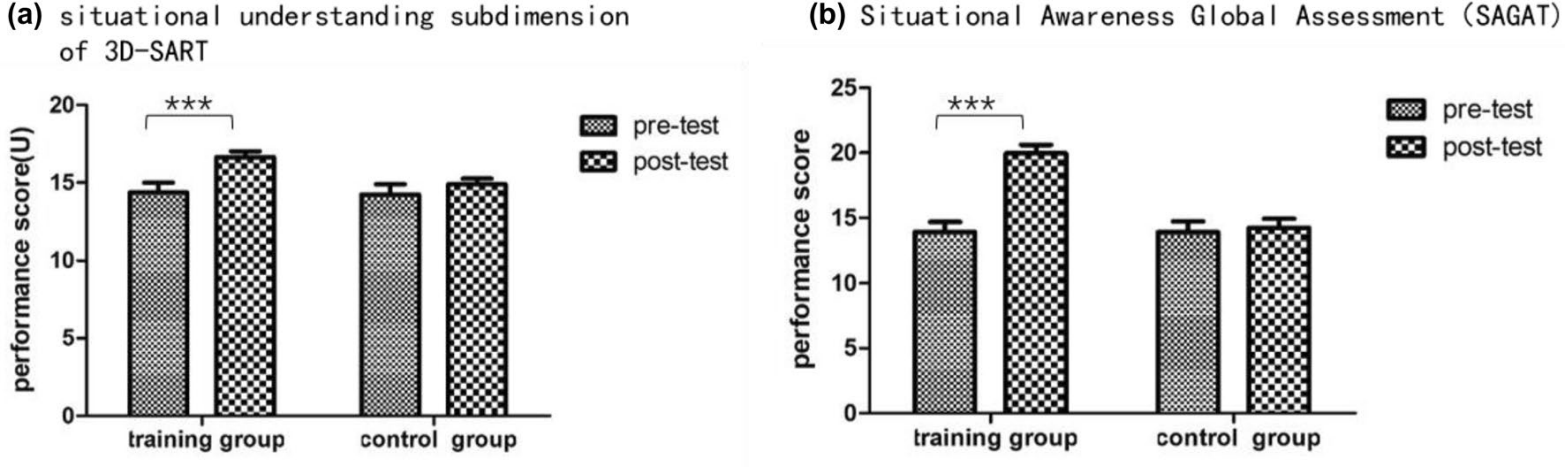
SA Performance of each group on the pretest and posttest. **A** Situational Understanding Subdimension Score of the 3D-SART. The graph depicts the Situational Understanding Scores. **B** SAGAT. The figure depicts Performance on the SAGAT

As shown in Table 1, there was no significant difference between the two groups in terms of pretest total scores [*t* (36) = 0.04, *P* = 0.967] or any of the three subdimension scores [*t* (36) = − 0.01, *P* = 0.990; *t* (36) = − 0.03, *P* = 0.977; *t* (36) = 0.14, *P* = 0.891] on the 3D-SART. First, a 2 (control group vs. training group) × 2 (pretest vs. posttest) mixed-model ANOVA of the total score of the 3D-SART revealed main effects of group (*p\P* = 0.400) and time (*P* = 0.052) as well as an interaction effect of time and group (*P* = 0.143). Then, mixed-model ANOVAs were used to evaluate the effect of training on each subdimension of the 3D-SART. The results for the attention demand subdimension showed main effects of time (*P* = 0.787) and group (*P* = 0.594) as well as an interaction effect (*P* = 0.347). The results for the attention supply subdimension showed main effects of time (*P* = 0.039) and group (*P* = 0.299) as well as an interaction effect (*P* = 0.097). The results for the situational understanding subdimension showed no significant main effect of group (*p* = 0.130), but the main effect of time [*F* (1, 36) = 13.70, *P* = 0.001, *η*^2^ = 0.28] and the interaction effect of group and time [*F* (1, 36) = 4.27, *P* = 0.046, *η*^2^ = 0.11] were significant. Through simple effect analysis, it was found that the situational understanding score in the training group was significantly higher after training than before training [*F* (1, 36) = 17.55, *P* < 0.001, *η*^2^ = 0.33], as shown in Fig. 4A, while no significant difference was found in the control group (*P* = 0.267). The results showed that working memory training improved the situational understanding subdimension score and thus improved subjective SA performance.

**Table 1:** Group mean (± SD) pretest and posttest scores on the 3D-SART and its three subdimensions

	Training group (*N* = 20)		Control group (*N* = 18)		*F*	*p*	*η*^2^
	Pretest	Posttest	Pretest	Posttest			
Total score	24.55 ± 8.26	28.35 ± 6.08	24.44 ± 7.39	25.00 ± 6.32	2.24	0.143	0.06
Attention demand (D)	9.15 ± 4.29	8.45 ± 3.17	9.16 ± 3.40	9.55 ± 3.67	0.91	0.347	0.03
Attention supply (S)	19.35 ± 4.23	20.15 ± 3.93	19.38 ± 4.17	19.67 ± 2.83	2.90	0.097	0.07
Situational understanding (U)	14.35 ± 2.97	16.65 ± 1.09	14.22 ± 2.69	14.89 ± 1.99	4.27	0.046	0.11

The statistical parameters presented in the table indicated a group × time interaction

### SAGAT results

There was no significant difference between the two groups in terms of SAGAT pretest scores [*t* (36) = 0.58, *P* = 0.581]. A 2 (control group and training group) × 2 (pretest and posttest) mixed-model ANOVA was performed for the test scores of the two groups, revealing significant main effects of group [*F* (1, 36) = 10.359, *P* = 0.003, *η*^2^ = 0.223] and time [*F* (1, 36) = 31.659, *P* < 0.001, *η*^2^ = 0.468] as well as an interaction effect of group and time [*F* (1, 36) = 25.392, *P* < 0.001, *η*^2^ = 0.414]. Then, the effects of the two factors were assessed through simple effect analysis, as shown in Fig. 4B. The score for the training group was significantly increased for the posttest compared with the pretest [*F* (1, 36) = 60.04, *P* < 0.001, *η*^2^ = 0.63], while there was no significant difference for the control group (*P* = 0.668). The results indicated that objective SA performance in the training group significantly increased after training compared with before training.

## Discussion

The current study mainly aimed to investigate the effect of working memory training on SA in a flight simulator to attempt to find a solution for improving SA. From the overall results of the experiment, it was found that working memory training not only improved performance on the dual N-back task but also, more importantly, increased SA.

After 2 weeks of continuous training, the performance of the training group on the dual *N*-back task gradually improved. The mean *N* level showed an upwards trend over the first nine training sessions and eventually stabilized in the subsequent sessions, clearly indicating a training effect. In addition, the training group showed greater improvements in fluid intelligence than the control group, which corroborates previous evidence that working memory training can indeed improve individuals’ cognitive abilities (Course-Choi et al. 2017; Li et al. 2021; Mattson et al. 2020; Miró-Padilla et al. 2020). Other studies failed to achieve transfer effects of training (Schwaighofer et al. 2015; Soveri et al. 2017; Vartanian et al. 2021), which may be because the differences were not significant due to an insufficient number of participants or because the training time and intensity did not reach the threshold needed to transfer learning between tasks (Kable et al. 2017).

This study found that the training group’s 3D-SART scores and SAGAT scores in the flight simulator were significantly increased after training, which indicated that working memory training effectively improved the participants’ SA during flight. Regarding the subjective questionnaire, its greatest advantage is that it is easy to apply, and can be readily adapted across different domains without modification, such as simulation-based studies and real scenario-based studies. However, it has also been criticized due to its subjectivity, as the subjective measurement of SA is likely to assess self-performance (Endsley 1995b), and furthermore, people's awareness of their own SA may be limited: people often are not aware of what they do not know (Endsley 1995b). This may be the reason why the difference between the two groups was not significant although the total posttest score of the training group exceeded that of the control group. To further analyze the impact of training on subjective assessments, three subdimensions of the questionnaire, attention demand, attention supply, and situational understanding, were analyzed and it was found that only in the situational understanding subdimension were the posttest scores of the training group significantly higher than those of the control group. In the two subdimensions of attention demand and attention supply, there was no significant difference between the two groups before or after training. On the one hand, one reason is that the flight scenario designed in the experiment was relatively simple and difficult to adapt and did not reach the upper limit of attention resources. On the other hand, all participants were young college students, whose basic cognitive abilities were relatively good, which produced a “ceiling effect”. In the situational understanding subdimension, the participants needed to temporarily store and process the perceived information on the basis of sufficient attention resources, which meant that the ability to understand the situation could be improved through training, so the situational understanding dimension score was significantly improved after working memory training (Endsley 2020).

In terms of objective methodology, the results showed that the SAGAT score of the training group increased significantly after the training compared with that of the control group, indicating that working memory training also had positive effects on SA during flight. Previous studies have confirmed the close relationship between working memory and SA as measured by the SAGAT (Cak et al. 2020; Endsley 2020; Gutzwiller and Clegg 2013). However, the underlying mechanisms of the relationship between working memory and SA are still unclear, and there has been insufficient empirical research on these mechanisms. The objective measurement results in this study are a helpful contribution to the field.

This study successfully observed that the beneficial effect of working memory training on SA may be affected by multiple factors. First, the information processing theoretical model proposed by Endsley (1995a) emphasizes that working memory is a key factor that restricts the improvement of SA. That is, good SA requires individuals to effectively allocate their attention, update various information in a timely manner, quickly switch task modes, suppress the interference of irrelevant information, etc. The dual *N*-back task chosen in this study involves multiple executive processes, including updating information representations, monitoring current stimuli, suppressing irrelevant stimuli, switching tasks quickly and allocating attention reasonably (Halford et al. 2007; Jaeggi et al. 2008; Salminen et al. 2016). Therefore, working memory training can improve participants’ SA. Second, a series of studies conducted by Klingberg et al. (2005) and Holmes et al. (2009) suggested that load adaptation and adequate training are two important factors that can transfer working memory training to other, untrained tasks. The term “load”, as used in this paper, refers to the maximum working memory capacity that an individual can reach. To a certain extent, the nervous system can adapt to the challenges of working memory tasks. When an individual continually works at a maximum load, it becomes relatively easy for them to perform under loads that initially require a great deal of effort. That is, through a self-adaptive training technique with adjustable difficulty, the maximum load can be constantly updated and eventually increased, reflecting the learner’s adaptation to the demands of the task. When the brain begins to adapt to a high volume of working memory training and shows a significant improvement in the training task, such as an increase in N of more than 2, transfer effects become more likely to occur. In general, the similar cognitive bases of working memory and SA, and the continuous adaptation of working memory capacity to match the current task load are two critical factors that enable working memory training to promote SA.

SA is closely related to safety. Previous studies have mostly focused on analyzing the influencing factors of SA from the perspective of accident analysis, to prevent the occurrence of similar accidents (Jones et al. 1996; Kharoufah et al. 2018). However, simply describing the phenomenon is not enough, and the cognitive mechanism of SA should be explored. In his information processing theory, Endsley (1995a) pointed out that SA is closely related to some important cognitive abilities, such as attention, working memory and long-term memory, indicating that cognitive training is an important target for improving SA. Actual research has indeed confirmed the improvement of SA through working memory training, which provides new ideas for future research in this field. SA is a research hotspot in the field of aviation safety and understanding its cognitive mechanism can help us to study and improve SA from the perspective of basic cognitive ability, and to conduct simpler and more direct assessment and training for pilots, which is also a major contribution of this study.

However, this study also has some shortcomings. First, long-term tracking was not feasible due to the COVID-19 pandemic; therefore, the duration of the transfer effect could not be determined. Second, further studies should use a variety of methods, for example, neuroimaging techniques can be used to find neurological evidence of the training effect (Feng and Delaney 2018) and physiological indicators to enrich SA measurement techniques (Vanderhaegen et al. 2020). Single scores from one or two approaches may not represent the complex, potentially intercorrelated elements of cognitive processing; therefore, it is necessary to use multiple techniques to assess SA in future research.

In conclusion, participants from the Air Force were selected as the research subjects to confirm the effectiveness of working memory training and its positive effect on SA in a complex operating environment. Throughout the experiment, various experimental conditions were strictly controlled, and the training and transfer effects were found to be statistically significant. The verified beneficial effect of working memory training on SA has important implications. Although the current research is only a laboratory study and needs to be followed by practical studies verifying ecological validity, its findings will provide useful guidance for future research on SA.
